# Further *In-vitro* Characterization of an Implantable Biosensor for Ethanol Monitoring in the Brain

**DOI:** 10.3390/s130709522

**Published:** 2013-07-23

**Authors:** Ottavio Secchi, Manuel Zinellu, Ylenia Spissu, Marco Pirisinu, Gianfranco Bazzu, Rossana Migheli, Maria Speranza Desole, Robert D. O′Neill, Pier Andrea Serra, Gaia Rocchitta

**Affiliations:** 1 Department of Clinical and Experimental Medicine, Medical School, University of Sassari, Viale S. Pietro 43/b, Sassari 07100, Italy; E-Mails: osecchi@uniss.it (O.S.); mzinellu@uniss.it (M.Z.); yspissu@uniss.it (Y.P.); mpirisinu@uniss.it (M.P.); gbazzu@uniss.it (G.B.); rmigheli@uniss.it (R.M.); desole@uniss.it (M.S.D.); paserra@uniss.it (P.A.S.); 2 UCD School of Chemistry and Chemical Biology, University College Dublin, Belfield, Dublin 4, Ireland; E-Mail: robert.oneill@ucd.ie

**Keywords:** ethanol biosensor, oxygen dependence, pH dependence, implantable biosensor

## Abstract

Ethyl alcohol may be considered one of the most widespread central nervous system (CNS) depressants in Western countries. Because of its toxicological and neurobiological implications, the detection of ethanol in brain extracellular fluid (ECF) is of great importance. In a previous study, we described the development and characterization of an implantable biosensor successfully used for the real-time detection of ethanol in the brain of freely-moving rats. The implanted biosensor, integrated in a low-cost telemetry system, was demonstrated to be a reliable device for the short-time monitoring of exogenous ethanol in brain ECF. In this paper we describe a further *in-vitro* characterization of the above-mentioned biosensor in terms of oxygen, pH and temperature dependence in order to complete its validation. With the aim of enhancing ethanol biosensor performance, different enzyme loadings were investigated in terms of apparent ethanol Michaelis-Menten kinetic parameters, *viz. I*_MAX_, *K*_M_ and linear region slope, as well as ascorbic acid interference shielding. The responses of biosensors were studied over a period of 28 days. The overall findings of the present study confirm the original biosensor configuration to be the best of those investigated for *in-vivo* applications up to one week after implantation.

## 1. Introduction

Ethanol is a ubiquitous psychoactive agent in Western society. Its effects are mainly associated with the modulation of GABAergic and glutamatergic systems; moreover, the positive reinforcing properties of ethanol are related to activation of dopaminergic pathways, producing dopamine release in the *nucleus accumbens*. In view of these neurobiological aspects, the detection of ethanol in brain extracellular fluid (ECF) is of great importance. Several studies in the literature have reported ethanol pharmacokinetics and concentrations in the brain after systemic injection, and showed that ethanol in the CNS could reach concentrations of about 30 mM [1–4]. In all those papers, neither “real-time” techniques nor implanted biosensors were used. In a previous paper [5], we introduced a biosensor suitable for monitoring ethanol concentration in brain in real time. In that study, different designs were investigated, all manufactured using a fixed concentration for enzyme stock solution (200 U·mL^−1^ of alcohol oxidase), and we also investigated the role of polyethyleneimine or glycerol (or a combination of both) as enzyme enhancers and stabilizers. In choosing the best design for *in-vivo* implantation, studies were also performed on the interference due to electroactive molecules present in brain ECF, mainly represented by ascorbic acid (AA). Starting from our previous findings, different designs of alcohol biosensors were developed and characterized in this study, using stock solutions of different enzyme concentrations. Again, the biological component, used as the sensing element, was the enzyme alcohol oxidase (AOx; EC 1.1.3.13), a flavoprotein with eight subunits, each containing a flavin adenine dinucleotide (FAD) cofactor molecule that plays a pivotal role in the enzyme activity (Reaction (1)). AOx is capable of catalyzing the oxidation of primary, aliphatic short-chain alcohols (such as ethanol and methanol) to their respective aldehydes as follows:

(1) $$\text{RC}{\text{H}}_{2}\text{OH}+\text{AOx}/\text{FAD}\to \text{RCHO}+\text{AOx}/{\text{FADH}}_{2}$$

(2) $$\text{AOx}/{\text{FADH}}_{2}+{\text{O}}_{2}\to \text{AOx}/\text{FAD}+{\text{H}}_{2}{\text{O}}_{2}$$

(3) $${\text{H}}_{2}{\text{O}}_{2}\to {\text{O}}_{2}+2{\text{H}}^{+}+2{\text{e}}^{-}$$

The hydrogen peroxide produced by Reaction (2) can be detected at a Pt surface by applying an anodic potential of +0.7 V *vs.* Ag/AgCl (Reaction (3)). Previously [6], the importance of oxygen interference in biosensor functionality has been described, which becomes particularly relevant in applications involving *in-vivo* monitoring, where pO_2_ can fluctuate significantly [7,8]. Thus, the suitability of the ethanol biosensor *in vivo* depends on the concentration of ethanol being monitored, as well as the range of fluctuations in local pO_2_ in the brain. As previously described, biosensor oxygen dependence can be measured as *K*_M_(O_2_) [9–11]. As *K*_M_(O_2_) defines, experimentally, the concentration of oxygen at which the biosensor signal, for a given concentration of analyte, is reduced to half [6]; a low value of *K*_M_(O_2_) implies a lower oxygen dependence, providing oxygen saturation of the enzyme at lower pO_2_. In this study we investigated the response of alcohol biosensors at a fixed ethanol concentration of about 15 mM, a value found *in vivo* after a single intragastric administration of 1 g·kg^−1^ of ethanol [5], as well as the biosensor response to varying the oxygen concentration in the electrochemical cell. In addition, the optimum working conditions of the biosensor with respect to pH and temperature were investigated.

## 2. Experimental Section

### 2.1. Chemicals

All chemicals were purchased from Sigma-Aldrich (Milano, Italy), unless stated otherwise. PBS (pH 7.4) was prepared by dissolving NaCl (8.9 g), NaOH (1.76 g), and NaH_2_PO4 (6.89 g) in 1 L of bidistilled water. Stock solutions of alcohol oxidase from *Hansenula polymorpha* (AOx, EC 1.1.3.13) were prepared in PBS, in a concentration range from 100 to 800 U·mL^−1^. Hydrogen peroxide (H_2_O_2_) stock solution (100 mM) was prepared in water by diluting the original 30% (w/w) solution. Ethanol solutions (1 M and 10 mM) were obtained from absolute ethanol by dilution in bidistilled water. Ascorbic acid solution (AA 100 mM) was prepared by dissolving L-ascorbic acid in 0.01 M HCl. Polyethyleneimine (PEI) and glycerol (Glyc) solutions were obtained by diluting the stock solutions (50% w/v and 87% w/v respectively) in bidistilled water. *ortho*-Phenylenediamine monomer solution (oPD, 300 mM) was prepared in deoxygenated PBS. Special caution is needed when using phenylenediamines; please refer to their respective material safety data literature. Polyurethane solution (PU, 1% w/v) was obtained dissolving PU beads in tetrahydrofuran (THF). Teflon®-coated platinum (90% Pt, 10% Ir; Ø = 125 μm) and silver wires (Ø = 250 μm) were purchased from Advent Research Materials (Eynsham, UK). Epoxy resin, Araldite-M and graphite were also purchased from Sigma-Aldrich. Ultrapure (>99.9%) oxygen and nitrogen were purchased from Sapio s.r.l. Special Gases Division (Caponago, Italy).

### 2.2. Ethanol Biosensor Fabrication, Ethanol and AA Response Studies

**Figure 1.**
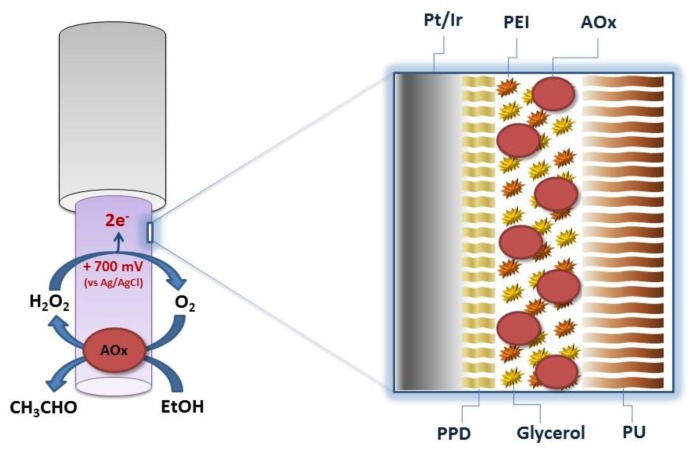
Schematic representation of the main design of implantable alcohol biosensors developed and characterized in this study. Pt_c_/PPD/[{PEI(1%)+Glyc(1%)}/AOx]10/PU(1%). Pt/Ir: 1 mm long, 125 μm diameter Pt/Ir cylinder; AOx: alcohol oxidase; PPD: poly-*ortho*-phenylenediamine; PU: polyurethane; PEI: polyethyleneimine. Different enzyme stock solutions were used, the concentration of which ranged from 100 to 800 U·mL^−1^.

Starting from a previously-developed biosensor for ethanol monitoring in brain [5], five new configurations were derived and manufactured. All biosensors were based on the same cylindrical geometry (1 mm length and 125 μm diameter of platinum wire) obtained by exposing the bare metal by cutting away the Teflon insulation, using a new sharp scalpel blade. The length of the exposed cylinder was trimmed to 1 mm (±5%), using an optical microscope. The main strategy used for increasing biosensor substrate sensitivity was varying the enzyme loading in each configuration. All designs had in common the same strategy for blocking AA interference: the electro-deposition of a poly-*ortho*-phenylenediamine (PPD) nanometer-thick film [12]. The PPD electrosynthesis was performed as follows: stock solutions of oPD monomer (300 mM) were freshly prepared in nitrogen-saturated PBS immediately before electropolymerization, which was carried out amperometrically at +0.7 V versus Ag/AgCl for 15 min. The deposition of the PPD always occurred on bare metal and then the AOx was loaded by means of 10 quick dips, from different enzyme stock solutions, each preceded by a dip in a mixed solution of enzyme stabilizers and enhancers (1% PEI and 1% glycerol). Finally, the biosensor was dipped in a 1% PU solution in order to entrap enzyme molecules (Figure 1). Slightly modifying the previous procedure, an enzyme-free sensor was built and used as reference for H_2_O_2_ calibrations as described below in the text.

As previously described [5], all biosensor configurations were characterized *in vitro* for ethanol response and AA interference blocking. The electrochemical studies were performed in a cell consisting of four biosensors as working electrodes, an Ag/AgCl (NaCl, 3 M) electrode as reference electrode, and a large surface-area Pt wire as auxiliary electrode. Both electropolymerization and calibrations were performed by applying a constant potential of +0.7 V versus Ag/AgCl, using the four-channel equipment (eDAQ QuadStat, e-Corder 410, eDAQ, Denistone East, Australia). The *in-vitro* response to ethanol was assessed by means of a full calibration (0-120 mM), performed with successive injections of known volumes of freshly-prepared ethanol stock solutions (10 mM and 1 M) in 20 mL of PBS at room temperature (22 ± 2 °C). Injections of 0.5 mM and 1 mM of AA were made in fresh PBS, in order to assess the blocking capability of the biosensor against AA as the main interference species present in rat brain [13]. The sensitivity to H_2_O_2_ was evaluated by studying the biosensor responses in a range comprised between 0 and 0.1 mM and comparing the results with those obtained with enzyme-free sensors.

In order to determine the stability of biosensors over time, a study on aging was conducted on all the designs: apparent Michaelis-Menten kinetic parameters (*I*_MAX_ and *K*_M_), sensitivity (LRS), 1 mM AA current and Δ*I* were monitored *in vitro* from day 0 (when biosensors were made and calibrated) up to day 28. When not undergoing calibration, the biosensors were stored in air at 4 °C in a fridge (following thorough rinsing with deionized water) during the entire period of the study.

### 2.3. Oxygen Sensor Fabrication and Calibration

The O_2_ sensors were prepared as previously described [14]. Approximately 1 mm of a Teflon-insulated silver wire (30 mm in length; i.d. Ø 125 μm, Advent Research Materials, Suffolk, UK) was exposed and inserted into a silica capillary tube (10 mm in length; i.d. 180 μm, Polymicro Technologies, Phoenix, AZ, USA) and partly filled with graphite-loaded (55% w/w) epoxy resin (Araldite-M, Sigma-Aldrich). By mixing 850 mg of graphite with 500 mg of Araldite-M and 200 mg of hardener [15], and filling the silica capillary tubing with the mixture, a preliminary 180 μm diameter carbon-composite disk electrode (area = 2.5 × 10^−4^ cm^2^) was fabricated and the silver wire was suitable for guaranteeing a good electrical contact. After 24 h at 40 °C, the electrode was given a conical shape by means of a high speed drill Dremel 300 equipped with an aluminum oxide grinding wheel, giving to the O_2_ microsensor a length ≈250 μm, a surface ≈1.5 × 10^−3^ cm^2^, and a tip <25 μm. Afterwards, a cellulose nitrate treatment was performed by immersing the carbon-composite disk in the collodion solution three times and drying it for 60 min after each coat at 40 °C. Constant potential amperometry (CPA) was used for *in-vitro* calibrations and experiments, fixing the O_2_ reduction potential at −0.4 V *vs.* Ag/AgCl reference electrode. Oxygen sensors calibration was performed after having immersed the sensors in fresh PBS, previously completely saturated with nitrogen (N_2_ 100%). After the stability of the baseline current was reached, different aliquots of an oxygen saturated solution (O_2_ 100%) were added in order to obtain an oxygen concentration ranging from 0 to 260 μM in the electrochemical cell, as previously described [14].

### 2.4. Oxygen Dependence Study Setup

In order to monitor the dissolved oxygen in the electrochemical cell, during oxygen dependence experiments, the previously described [14] conical-shaped sensors were used. All experiments were performed in a standard three-electrode gas-tight electrochemical cell containing 20 mL PBS at room temperature. Two different potentials were applied: three ethanol biosensors were polarized at +0.7 V *vs.* Ag/AgCl, while the oxygen microsensor was polarized at -0.4 V *vs.* Ag/AgCl. At the beginning of experiments, sensors were immersed in PBS completely saturated with nitrogen (N_2_ 100%) containing 15 mM ethanol and then polarized. After having reached current stability, different aliquots of an oxygen saturated PBS solution (O_2_ 100%), also containing 15 mM of ethanol, were added, ranging from 0 to 260 μM. The presence of the oxygen microsensor was required to verify the oxygen concentration in the electrochemical cell.

### 2.5. pH and Temperature Dependence Study Setup

The effect of pH on the ethanol biosensor response was examined at room temperature in the presence of a fixed ethanol concentration. The study was performed in a pH range comprised between 6.0 and 9.8, since it is well known that the functional pH region of AOx extracted from *Hansenula* sp. ranges from 5.5 to 8.5, as previously described [16]. The most suitable design for *in-vivo* implantation (selected on the basis of the oxygen dependence results) was chosen and it was exposed to the fixed concentration of 15 mM ethanol, dissolved each time in fresh PBS at different pH. The relative current obtained from each experiment was then plotted (as shown in Figure 5 later). The temperature dependence was determined for 15 mM EtOH at physiological pH (7.4) in a range comprised between 20 and 40 °C.

### 2.6. Statistical Analysis

Currents were expressed in nanoampere (nA) and given as baseline-subtracted values ± standard error of the mean (nA ± SEM). The AA ΔI value represents the difference between the current resulting from the injection of 1 mM and 0.5 mM of AA in the electrochemical cell, as discussed previously [17]. Limits of detection and quantification (LOD and LOQ, Equations (4) and (5)) were determined using a statistical method based on the standard deviation ( *σ* ) of the response and the linear region slope (LRS) of the calibration curve [18] as follows:

(4) LOD=3.3σ/LRS

(5) LOQ=10σ/LRS

Variations in hydrogen peroxide sensitivity were calculated as percentage changes compared to enzyme-free sensors responses (100%). Statistical significance (*P* values) between groups was evaluated using unpaired *t*-tests. Concentrations of dissolved O_2_ were expressed in micromoles per liter while the oxygen reduction current was expressed in nA ± SEM. The sign of the oxygen currents (cathodic) was inverted to improve the readability of these data.

## 3. Results

### 3.1. Effects of Enzyme Loading on Biosensor Michaelis-Menten Kinetics, Ethanol Sensitivity and AA Response

In order to enhance ethanol biosensor performance, different enzyme loadings were investigated in terms of apparent ethanol Michaelis-Menten kinetic parameters, *I*_MAX_ and *K*_M_, the LRS and enzyme oxygen dependence. In all designs, 10 quick dips were performed using different enzyme solutions, the concentration of which ranged from 100 to 800 U·mL^−1^.

As shown in Table 1, *I*_MAX_ increased almost linearly with the increase of the enzyme loading, ranging between 75 ± 6 and 308 ± 10 nA when the enzyme concentration was varied from 100 to 400 U·mL^−1^ (0.80 ± 0.14 nA·U^−1^·mL; *R*^2^ = 0.975; *n* = 4). *I*_MAX_ values tended to exponentially decay (*R*^2^ = 0.983; *n* = 4) when the enzyme loading solution varied from 400 up to 800 mL^−1^ with a halving of the current every 57 U·mL^−1^ and a plateau of 44 ± 13 nA. This was unexpected, and was due in part to a decrease in H_2_O_2_ sensitivity (see Table 2 and discussion below). Relative to *K*_M_, linear increases were observed up to 400 U·mL^−1^ (0.074 ± 0.009 mM·U^−1^·mL; *R*^2^ = 0.987; *n* = 20) while no relevant differences were found increasing enzyme loading further. The LRS tended to increase in parallel to the concentration of enzyme in solution up to 400 U·mL^−1^ (0.010 ± 0.002 nA·mM^−1^·U^−1^ mL; *R*^2^ = 0.989; *n* = 20), reaching the maximum value of 3.94 ± 0.12 nA·mM^−1^. The further increase in enzyme concentration produced an exponential decay in the sensor response to ethanol (*R*^2^ = 0.981; *n* = 20) with a halving of the resulting current every 56 U·mL^−1^ of AOx and a plateau of 0.65 ± 0.15 nA·mM^−1^. The linear region of the biosensor response was observed between 0 and 40 mM for all studied groups except for the 100 U group that showed a smaller concentration span (0–30 mM).

Increasing enzyme loading also influenced the LOD and LOQ as illustrated in Table 2. LOD values increased proportionally to the concentration of enzyme, ranging from 0.006 ± 0.004 mM (LOQ = 0.2 ± 0.1 mM) to 0.053 ± 0.014 mM (LOQ = 0.42 ± 0.19 mM).

Hydrogen peroxide sensitivity decreased with enzyme loading in a linear manner (–5.6 ± 0.4% for every 100 U·mL^−1^ increase of AOx in the dipping solution; *R*^2^ = 0.976; *n* = 20). Ascorbic acid interference, measured at day 1, was similar for all studied groups (Table 2); after the exposure of the biosensors to a standard concentration of AA (1 mM), the recorded currents ranged from a minimum of 0.98 ± 0.27 nA (400 U·mL^−1^) to a maximum of 1.03 ± 0.29 (800 U·mL^−1^).

**Figure 2.**
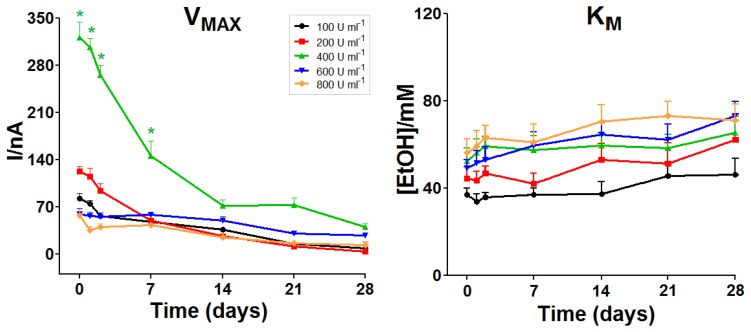
*In-vitro* stability study: evolution of the enzyme kinetic parameters, *I*_MAX_ and *K*_M_, over a 28-day monitoring period, for biosensors fabricated from different enzyme loading solutions (*n* = 4 for each group). * *p* < 0.05 *vs.* other groups.

The responses of biosensors over time were assessed over a period from day 0 up to day 28. *I*_MAX_, *K*_M_, LRS, AA ΔI and AA 1 mM current (data not shown) were monitored *in vitro* as described in Section 2.2. Unsurprisingly, all the selected designs showed a global, rather homogeneous, decay in terms of *I*_MAX_ (Figure 2, left panel). However, only the design made with the 400 U·mL^−1^ enzyme solution showed *I*_MAX_ values higher to other designs, remaining so up to day 21.

**Figure 3.**
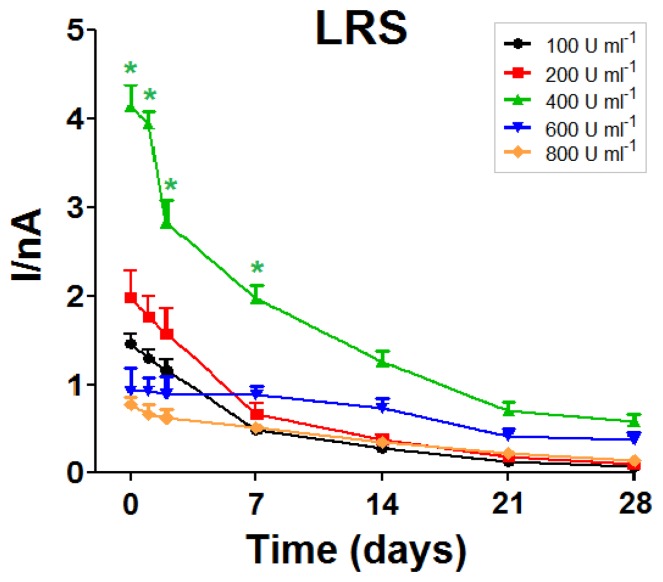
*In-vitro* stability study: evolution of the LRS over a 28-day monitoring period for biosensors fabricated from different enzyme loading solutions (*n* = 4 for each group). * *p* < 0.05 *vs.* other groups.

Mean values of *K*_M_ were found to be not significantly proportional to enzyme loading solution up to 800 U·mL^−1^ (Figure 2, right panel). Over time, *K*_M_ values showed a rather homogeneous behavior for all designs, tending to slowly increase over the whole monitoring period. As illustrated in Figure 3, observed LRS values and trends were similar to those previously described above for *I*_MAX_. Just like *I*_MAX_, the LRS of the biosensors made with the 400 U·mL^−1^ solution was higher than for other designs.

**Figure 4.**
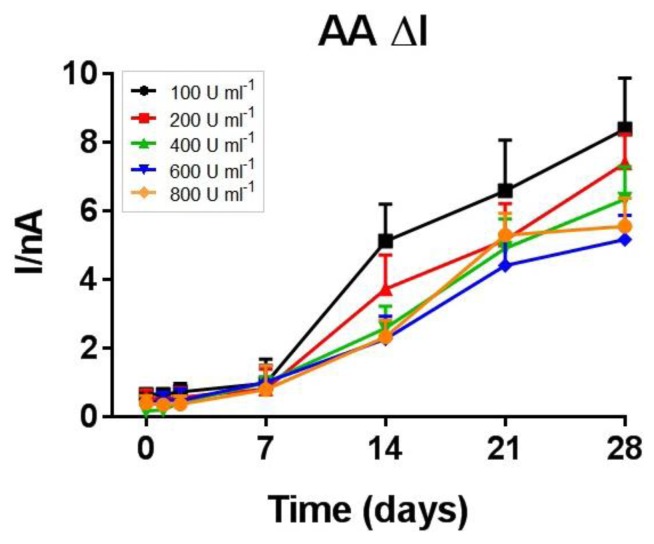
*In-vitro* stability study: evolution of the AA ΔI, over a 28 days monitoring period for biosensors fabricated from different enzyme loading solutions (*n* = 4 for each group).

Ascorbic acid interference, measured during the entire 28-day period and expressed as AA ΔI (Figure 4), was similar in all studied groups up to day 7 (0.47 ± 0.04 nA; *n* = 20); after the first week all the studied groups showed an increasing sensitivity to AA with an observed slope between 0.21 ± 0.03 nA day^−1^ (600 U·mL^−1^) and 0.34 ± 0.06 nA day^−1^ (100 U·mL^−1^).

### 3.2. Oxygen and Temperature Dependence of Biosensor Responses

As shown in Table 3, oxygen *I*_MAX_ increased almost linearly with the increase of the number of AOx molecules, ranging between 19.1 ± 3.5 and 64.2 ± 4.3 nA when the enzyme concentration was varied from 100 to 400 U·mL^−1^ (0.16 ± 0.03 nA·U^−1^ ·mL; *R*^2^ = 0.974; *n* = 20). There was a parallel increase in oxygen dependence on the biosensor response (*K_M_* for O_2_). *I*_MAX_ values tended to exponentially decay (*R^2^* = 0.924; *n* = 20) when the enzyme loading solution varied from 400 up to 800 U·mL^−1^ with a halving of the current every 55 U·mL^−1^ and a plateau of 13.4 ± 4.6 nA. Surprisingly, the oxygen dependence continued to increase for these biosensors. Overall, a linear increase in oxygen dependence (*K_M_*(O_2_) = 0.091 ± 0.003 μM·U^−1^mL; *R*^2^ = 0.997; *n* = 20) was observed with the increase of the enzyme concentration in the loading solution.

### 3.3. pH and Temperature Dependence on Biosensor Response

**Figure 5.**
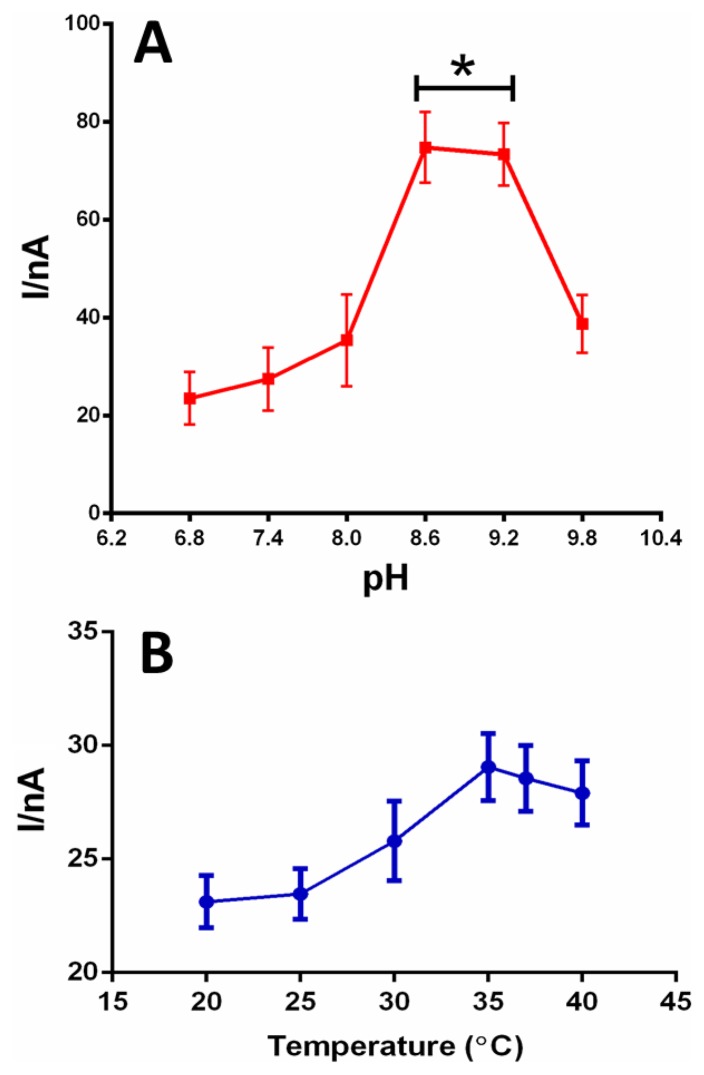
The influence of pH (A) and temperature (B) on the response of the biosensor Pt_c_/PPD/[{PEI(1%)+Glyc (1%)}/AOx]10/PU(1%), with 200 U·mL^−1^ of enzyme loading solution, exposed to a 15 mM ethanol concentration. * *p* < 0.05 *vs.* pH 6.8–8.0.

In Figure 5A, the study of the influence of the pH on the best biosensor design suitable for implantation (200 U·mL^−1^ of AOx, even selected on the basis of the good oxygen dependence results) is shown. We investigated the response of the biosensor to varying the pH at a constant concentration of about 15 mM (value found *in vivo* after a single administration of 1 g·kg^−1^ of ethanol i.g.) [5]. The results showed a clear increase in the analytical signal from pH 6.8 up to 8.0, but with no significant differences The maximum biosensor response (about 300% higher than at pH 7.4) was obtained between pH 8.6 and 9.2 (*p* < 0.05 *vs.* pH 6.8–8.0); above this value the response of the biosensor returned down to pH 8.0 levels.

The temperature dependence of surface-bound AOx was determined for 15 mM EtOH at physiological pH (7.4), using the Ptc/PPD/[{PEI(1%)+Glyc(1%)}/AOx]10/PU(1%) biosensor design. Although some temperature dependence was observed (Figure 5B), this was considerably less than that observed for the enzyme in solution [19]. Specifically, the biosensor response was only 18% less at 20 °C compared with the maximum responses observed between 35 and 40 °C. Thus, the difference in oxygen demand at the two temperatures is not likely to have a major impact on the biosensors′ oxygen tolerance *in vivo* (37 °C).

## 4. Discussion and Conclusions

The aim of the present study was to characterize further *in vitro* a previously-developed amperometric biosensor capable of monitoring ethanol concentration changes in brain ECF [5]. Varying the amount of enzyme in the loading solution (from 100 to 800 U·mL^−1^ of AOx), several related alcohol biosensor designs were developed and characterized in terms of *I*_MAX_, *K*_M_, LRS, and AA shielding power *in vitro*. These parameters are extremely important for evaluating the responses to ethanol and the main interference species. The oxygen-, temperature- and pH-dependence studies complete the *in vitro* biosensor characterization in order to choose the best implantable design. *I*_MAX_ is a measure of the amount of active enzyme molecules on related biosensor surfaces provided the sensitivity to hydrogen peroxide is known [12,20]. *I*_MAX_ increased linearly with enzyme loading in a range comprised between 100 and 400 U·mL^−1^. The highest *I*_MAX_ values (308 ± 10 nA) were obtained with an AOx loading solution of 400 U·mL^−1^; after this peak, the response exponentially decreased. A similar trend was observed for LRS (Table 1). It is surprising to observe this decrease in ethanol response for the more concentrated enzyme solutions. This phenomenon could be explained by the reduction of the number of active enzyme molecules on the biosensor surface. This hypothesis is not consistent with oxygen dependence results: the fact that the *K*_M_(O_2_) increased monotonically as the concentration of enzyme in solution increased, suggests that the loading of active enzyme is also increasing monotonically and producing more H_2_O_2_. An alternative explanation is that the excessive loading of macromolecules on the surface has decreased the electrodes′ sensitivity to H_2_O_2_[21], as confirmed by the hydrogen peroxide calibration results showed in Table 2.

Even the presence of PEI and glycerol may affect the response of AOx as previously described [5]. In brief, PEI led to an increase in *I*_MAX_ and LRS (acting as an “enzyme activity enhancer”) [22] while glycerol gave greater stability to the biosensor over time. As known, PEI is a positively-charged molecule that interacts with the negative charges of the oxidase enzymes (at pH 7.4) [22] enhancing their catalytic activity. It is possible that the best combination between enzyme loading and PEI enhancement has been obtained at 400 U·mL^−1^ and a further increase in enzyme loading has unbalanced this equilibrium.

The apparent *K*_M_ is the substrate concentration that gives half the *I*_MAX_ response [23]. In biosensor design, *K*_M_ has a dual importance: it determines the amplitude of linear region slope of the biosensor substrate (LRS = *I*_MAX_/*K*_M_), as well as the concentration range of the linear response (∼½ *K*_M_) [12,22]; thus, the higher the *K*_M_ value, the smaller is the LRS but the wider is the linear response region. All the approaches used here aimed to increase as much as possible *I*_MAX_ and *K*_M_ values in order to achieve the highest ethanol LRS and linear range. Even though LRS is generally valid only up to half the *K*_M_ value [12,20], our studied biosensors showed a good linearity up to 40 mM with *R*^2^ > 0.996 (Table 1) because of slight deviations from Michaelis-Menten behavior (*R*^2^ < 0.984, Table 1) in the form of a flattening of the calibration plot at low concentrations. In our experiments, *K*_M_ increased linearly with enzyme loading up to 400 U·mL^−1^, remaining at stable values for higher loads. This trend reflects both a decrease of the affinity of the enzyme to substrate and an increasing difficulty of the substrate to access to enzyme catalytic sites [23]. The above-discussed enzyme–PEI unbalanced equilibrium and an increase of the enzyme molecules on the biosensor surface could be responsible of the *K*_M_ increase. This observation is confirmed by the progressive increase of LOD and LOQ values with the enzyme loading.

The 28-day study demonstrated that *I*_MAX_ and LRS values decreased over time while *K*_M_ slowly increased. The configuration with 400 U·mL^−1^ AOx expressed *I*_MAX_ and LRS values significantly higher than the others during the first week. These biosensors, however, showed a significant oxygen dependence (see below), leaving the 200 U·mL^−1^ AOx configuration as the most sensitive one for use in oxygen-challenged media.

All biosensor designs showed a good AA shielding power with a range of values between 0.98 and 1.73 nA for 1 mM AA on day 1 (Table 2) and an averaged value of 0.47 ± 0.04 nA for AA ΔI during the first 7 days. After the first week, all the studied groups showed an increasing sensitivity to AA confirming that these biosensors cannot be implanted chronically for more than one week because of the decrease in their selectivity associated with the increase in AA interference [5]. Although not statistically significant, the AA results show that the increase in enzyme loading progressively improves the shielding performance against AA. The above-hypothesized loss of positive charges and an increase in negative charges could justify this trend as AA is anionic at pH 7.4.

The oxygen-dependence studies showed that *I*_MAX_(O_2_) increased with enzyme loading up to 400 U·mL^−1^ (64.2 ± 4.3 nA). The higher *I*_MA_X (O_2_) values were obtained with a load of AOx corresponding to 400 U·mL^−1^; after this peak, the response exponentially decreased. This trend reflects the above-discussed response to ethanol. The apparent *K*_m_(O_2_) increased linearly with enzyme loading reaching the value of 72 ± 11 μM at 800 U·mL^−1^. Dissolved oxygen concentrations in the brain (striatum), calculated in a previous study [14] corresponded to about 37 μM, a value consistent with other results from international literature [24-26]. Twice the apparent *K*_M_(O_2_) value corresponds to the oxygen concentration in enzyme saturation conditions (*I*_MAX_(O_2_)). As a result of this, for monitoring ethanol concentrations in the brain ECF independently from oxygen physiological oscillations, it is necessary that the biosensor *K*_M_(O_2_) has to be lower than one half the oxygen concentration dissolved in the brain ECF (<18 μM in relation with our previous findings). As illustrated in Table 3, only biosensors with enzyme loading up to 200 U·mL^−1^ fulfill this requirement.

The pH-dependence results demonstrated that the enzyme exhibited quite a stable response at physiological concentrations of H^+^ (in the brain) in a range comprised between pH 7.36 and 7.42; pH values between 8.6 and 9.2 resulted in the best biosensor performance in agreement with previous studies [16].

As result of the overall findings of the present study, the original design (200 U·mL^−1^ AOx) has been confirmed to be the best biosensor for *in-vivo* studies up to one week after implantation. The previous determination of fast response times to ethanol (<2 s) for this configuration [5] is also a useful property in this context. Although the biosensor made with 400 U·mL^−1^ has proved to have the best performances, in terms of *I*_MAX_ and LRS slope, it appears to have an excessive oxygen dependence compared to the concentrations of dissolved oxygen in the brain ECF. This biosensor design could be suitable for *in-vivo* experiments when the concentrations of ethanol are not particularly high: for example in self-administration experiments, where the peaks of ethanol are not so pronounced and the consumption of oxygen is not pushed to the extreme. Moreover, we suggest that the biosensor made with 400 U·mL^−1^ could be the best design for *in-vitro* experiments, in terms of its global performance.

## Figures and Tables

**Table 1.** Biosensor ethanol response study. *In-vitro* characterization at Day 1 (*n* = 4 for each group) of different biosensor designs with different enzyme loading. Table shows apparent Michaelis-Menten kinetic parameters (*I*_MAX_ and *K*_M_) and linear region slope (LRS) in the reference linear range.

**AOx Loading Solution (U**·**mL^1^)**	**Michaelis–Menten Kinetics**			**Linear Regression**		
	
	**EtOH*I*_MAX_(nA)**	**EtOH*K*_M_(mM)**	***R*^2^**	**EtOH Concentration Limit (mM)**	**EtOH LRS (nA**·**mM^1^)**	***R*^2^**
**100**	75 ± 6	34 ± 7	0.974	30	1.09 ± 0.06	0.996
**200**	114 ± 7	44 ± 4	0.984	40	1.77 ± 0.08	0.997
**400**	308 ± 10	56 ± 6	0.981	40	3.94 ± 0.12	0.996
**600**	67 ± 13	52 ± 11	0.976	40	0.92 ± 0.09	0.995
**800**	46 ± 10	59 ± 9	0.983	40	0.67 ± 0.15	0.991

**Table 2.** *In-vitro* characterization of different biosensor configurations, with different enzyme loadings, at Day 1 (*n* = 4 for each group) in terms of LOD and LOQ (Equations (4) and (5)), H_2_O_2_ sensitivity decrease, and 1 mM AA interference.

**AOx Loading Solution (U mL^−1^)**	**LOD ± SEM (μmol L^−1^)**	**LOQ ± SEM (μmol L^−1^)**	**H_2_O_2_ Sensitivity Drop (%*vs.* AOx-Free Design)**	**1 mM AA (nA)**
**100**	6 ± 4	20 ± 6	−10 ± 5%	1.32 ± 0.31
**200**	11 ± 3	30 ± 10	−19 ± 4%	1.73 ± 0.13
**400**	17 ± 6	56 ± 16	−28 ± 5%	0.98 ± 0.27
**600**	32 ± 18	106 ± 31	−39 ± 5%	1.64 ± 0.42
**800**	53 ± 14	164 ± 59	−46 ± 5%	1.03 ± 0.29

**Table 3.** Oxygen dependence study at Day 1. *In-vitro* characterization of the same biosensor groups shown in Tables 1 and 2.

**AOx Loading Solution (U**·**mL^1^)**	**Apparent Michaelis-Menten Kinetic Parameters for Oxygen**		
	**O_2_*I*_MAX_(nA)**	**O_2_*K_M_*(μM)**	***R*^2^**
**100**	19.1 ± 3.5	11.1 ± 2.3	0.982
**200**	27.4 ± 5.2	17.3 ± 4.4	0.978
**400**	64.2 ± 4.3	37.7 ± 5.1	0.989
**600**	17.4 ± 3.4	57.2 ± 8.3	0.977
**800**	13.7 ± 3.7	72.3 ± 11.2	0.972
